# Axial and rotational alignment of lower limb in a Caucasian aged non-arthritic cohort

**DOI:** 10.1007/s00590-020-02763-7

**Published:** 2020-08-12

**Authors:** L. Farinelli, M. Baldini, A. Bucci, S. Ulisse, F. Carle, A. Gigante

**Affiliations:** 1 Clinical Orthopaedics, Department of Clinical and Molecular Sciences, Università Politecnica Delle Marche, Ancona, Italy; 2 Centre of Epidemiology, Biostatistics and Medical Information Technology, Università Politecnica Delle Marche, Ancona, Italy; 3 Department of Radiological Sciences, Università Politecnica Delle Marche, Ancona, Italy

**Keywords:** Lower limb, Alignment, Rotational, Aged non-arthritic cohort

## Abstract

**Background:**

The alignment of human lower limb has been an area of ongoing study for decades. The purpose of this study was to analyze the axial and rotational alignment from hip to ankle in a Caucasian aged non-arthritic cohort.

**Methods:**

A non-arthritic cohort of aged patients was retrospectively analyzed by computer tomography. Anatomical–mechanical angle of femur (AMA), femur inclination (FI), femoral anteversion (FA), posterior condylar angle (PCA), proximal tibial torsion (TEAs-PTC and TEAs-PTT) and tibial fibular torsion (PTC-TFA) were measured.

**Results:**

The median age of the patients was 76 years (range 67 to 91 years). Regarding axial alignment, the AMA was 5 (2.94; 6.80). No significance differences were reported by side and age. AMA was significantly lower in men. The FI was 125.3 (120.0; 134.8) with no differences in terms of side, age or gender. Regarding torsion alignment, the median values of FA, PTC-TFA and TEAs-PTT were, respectively, 16.8, 28.5 and − 1.4. No differences were reported by age. Right tibia was externally rotated by 1.5 degrees as compared to the left side (P 0.035).

**Conclusion:**

The broad variability of the parameters analyzed highlights the necessity for a more anatomical and individualized approach during surgery of lower limb. The present study offers the fundament to understand and treat lower limb deformities. Hence, these data can constitute the normal reference values useful to investigate lower limb malalignment. Moreover, it helps to assess the possible changes of axial and rotational alignment in idiopathic OA of lower limb.

**Level of evidence III:**

Retrospective cohort study

## Introduction

The alignment of human lower limb has been an area of ongoing study for decades. However, a clear definition of “normal axial and rotational alignment” in non-arthritic adults has not been established yet. Any femur, patella and tibia axial and rotational malalignment might have a direct effect on the load transmitted through the joint leading to increase cartilage wear and degeneration [1]. Knee surgeons learned from total knee arthroplasty (TKA) procedures that a malposition of implants due to axial and/or rotational malalignment can result in higher revision rates and lower patient-reported outcome scores [2–4]. Likewise, it is conceivable that the axial and rotational alignment of lower limb may be crucial for the biomechanics of native hip knee and ankle. The deleterious effect of axial malalignment (i.e., valgus and varus deformities) of hip, knee and ankle joints is well recognized in the developing of osteoarthritis (OA) [5]. On the other hand, the pathological role of torsion malalignment of lower limb is relatively neglected [6]. Indeed, only few studies reported that a tibia torsion malalignment might be responsible for patellofemoral instability, medial gonarthrosis, patellar chondromalacia and Osgood–Schlatter disease [7–10]. Regarding the question of what constitutes “normal” alignment, there have been detailed studies of apparently healthy populations [11, 12]. Unfortunately, these studies have several limitations. Firstly, they are carried out with the use of plain radiographs considering only deformities in the coronal plane. Secondly, they included a wide age range of population where some patients might be too young to have idiopathic OA. Therefore, the role of rotational alignment in healthy and pathological conditions deserves to be deepened. The purpose of the present study was to analyze the axial and rotational alignment from hip to ankle in a Caucasian aged non-arthritic cohort.

## Material and methods

**Fig. 1:**
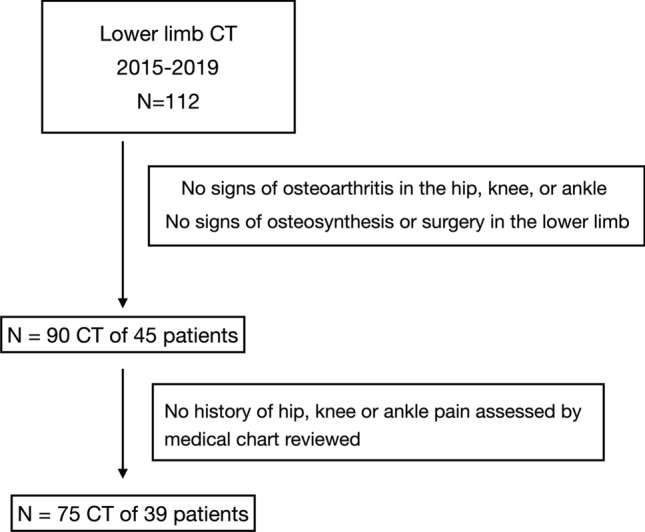
Patient flowchart

**Fig. 2:**
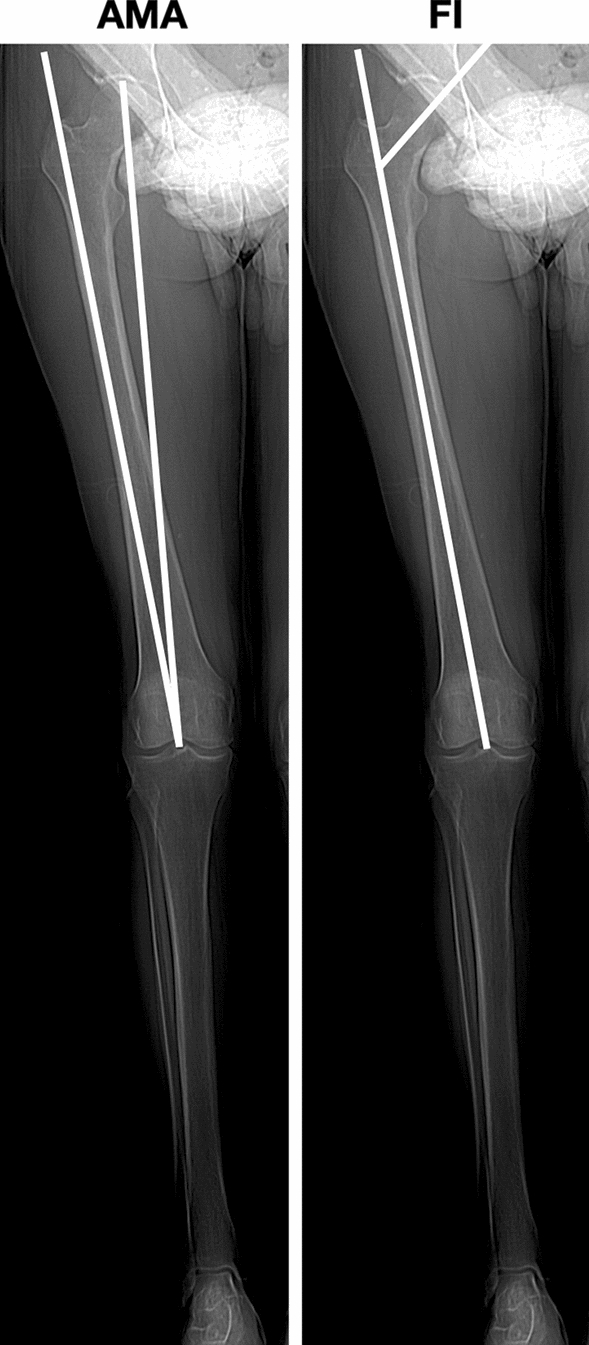
Axial alignment of lower limb: lines and angles analyzed in the present study. *AMA* anatomical–mechanical angle, *FI* femoral inclination

**Fig. 3:**
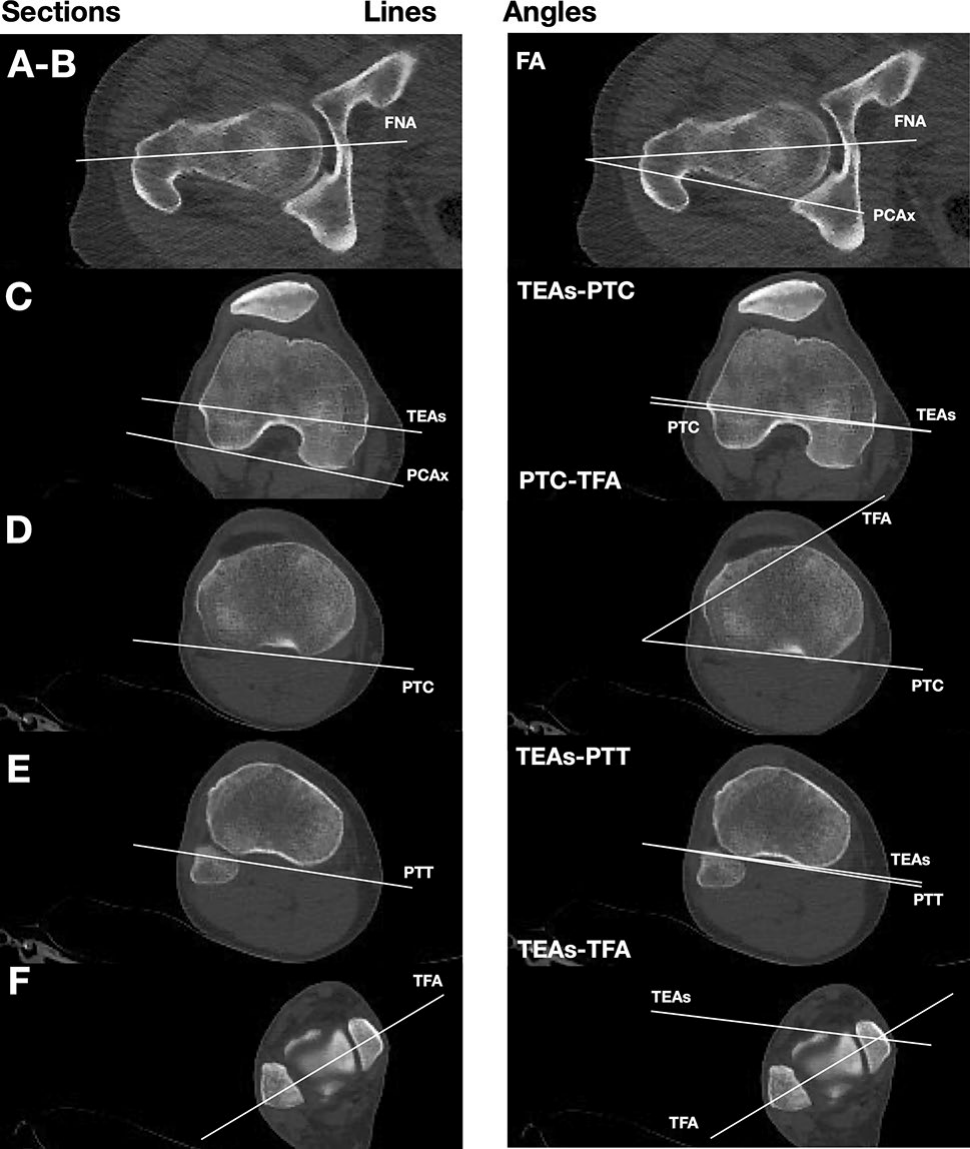
Torsion alignment of the lower limb: lines and angles analyzed in the present study. From the scout view, we find the following CT sections as explained in the text (**a**–**f**)

The study protocol was approved by the institutional ethics review board at our institution. A total of 115 full lower extremity computer tomography (CT) studies for oncological diseases workup were performed at our hospital between January 1, 2015, and December 31, 2019. At January 2020, patients’ files and the hospital’s digital database were reviewed retrospectively. A study flowchart is shown in Fig. 1. All scans were obtained using the same instrumentation (Pace General Electric, 4 s scanning time, 512 × 512 reconstruction matrix, 2 mm slice thickness, 125 kV, 460 mAs, with high resolution). All measurements were taken using the same software (Centricity Web V3.0, GE Medical Systems Information Technologies). Each CT examination required 6–9 sections [13]. CT sections were taken: (A) at the center of the femoral head; (B) at the base of the femoral neck; (C) in the midtrochlear region of the femoral condyle, identified by the Roman arch appearance of the intercondylar groove with the apex of the Roman arch corresponding to 1/3 of the height of the condyle [14]; (D) at the proximal tibial epiphyseal (PTE) level, which lies midway between the tibial plateau and the upper end of the proximal tibiofibular joint; (E) at the proximal end of the anterior tibial tuberosity (TT), (F) at the distal tibiofibular joint (TF). From the sections, these straight lines were delineated: a line passing through the center of the femoral head and neck, transepicondylar surgical axes (TEAs), posterior condylar axes (PCAx), a tangent to the most posterior prominent point of the medial and lateral tibial condyles (PTC) in PTE section; a tangent to the posterior surface of the tibia at TT sections (PTT), a line joining the center of both malleoli as described by Rosen et al. [15] (TFA). Three authors (LF, MB and SU) marked the lines on tomograms twice. The interval between those measurements was 4 weeks to avoid a possible memory effect. Once, the lines were established and the authors agreed, the angles were measured. The tomograms thus obtained were analyzed measuring: (1) anatomical–mechanical angle of femur (AMA) (Fig. 2); (2) femoral anteversion (FA) and inclination angles (FI); (3) posterior condylar angle (PCA) [16]; (4) TEAs-PTC angle; (5) TEAs-PTT angle; (6) TEAs-TFA angle; (7) PTC-TFA angle as shown in Fig. 3. A negative value was defined as internal torsion and a positive value as external torsion.

### Statistical analysis

We reported the median value, the 2.5th percentile and the 97.5th percentile for each tomograms measure considered. The following characteristics were considered to stratify the analysis: side of the limb, gender of the patient and age. In terms of age, patients were stratified in two classes: age equal or lower than 75 years and age over 75 years. We verified if the variables were normally distributed through Shapiro–Wilk test. We compared the distributions of variables through nonparametric tests because the variables were not normally distributed (except for FA). Specifically, we used Wilcoxon rank-test for paired samples when analyzing the variables between right and left limbs, while a Wilcoxon rank-test for independent sample was implemented to analyze the differences between men and women and age groups in each limb. Finally, we implemented a Friedman test [17] to multiple compare the relationship between the angles and the variables considered, i.e., side of the limb, gender and age group. *P* values less than 0.05 were interpreted as indicative of statistically significant differences. Statistical analyses were performed with the R statistical program [18].

## Results

A review of medical records at our institution identified 115 patients who underwent lower limb CT. At the end, 75 full lower extremity CT studies were included in the study; thirty-nine patients were included in the study, 36 with both limbs, 3 with a single limb. The median age of the patients was 76 years (range 67–91 years), while 19 (49%) patients were women. The summary statistics of the variables for both the limbs and stratified by side of the limb are reported in Table 1. The PTC-TFA angle was significantly higher in the right limb, while the FA was significantly higher in the left limb; no significant differences were observed in the other measures. None of the other measures showed a statistically significant difference between right and left limb. The comparison of tomograms measures by gender in each limb is shown in Table 2. There were no significant differences between males and females’ limbs, except for AMA, FA and PCA angles. The values of FA were significantly higher in female in the right limb, as well as the PCA values; AMA values were significantly higher in female both limbs. In Table 3, we compared the age groups stratifying by limb, and we found no statistically significant differences. Friedman’s test for multiple comparisons highlighted that variables considered affected somehow only the FA value (*p*. 0.008).

**Table 1:** Median and percentiles for each variable considered stratified by side of the limb

	Limb (*n* = 75)	Right Limb (*n* = 36^a^)	Left Limb (*n* = 36^a^)	*p* value
	Median (2.5%; 97.5%)	Median (2.5%; 97.5%)	Median (2.5%; 97.5%)	
FA	16.8 (1.0; 34.2)	12.3 (− 0.4; 36.0)	19.5 (1.6; 32.0)	0.010
PCA	3 (− 0.2; 6.2)	3 (− 0.1; 5.3)	3 (− 0.3; 5.6)	0.837
TEA-PTC	1 (− 6.6; 8.2)	1.8 (− 6.2; 9.1)	1.3 (− 6.7; 7.3)	0.661
TEA-PTT	− 1.4 (− 7.9; 4.2)	− 1.2 (− 7.2; 3.3)	− 1.75 (− 7.7; 4.4)	0.483
TEA-TFA	30.5 (12.5; 43.5)	31.4 (18.0; 43.5)	30.4 (9.2; 43.1)	0.142
PTC-TFA	28.5 (12.0; 45.4)	29.4 (17.5; 44.2)	27.9 (11.4; 45.3)	0.035
AMA	5 (2.9; 6.8)	5 (3.0; 6.8)	5 (2.9; 6.4)	0.606
FI	125.3 (120.0; 134.8)	125.1 (119.8; 134.3)	125.5 (121.5; 134.8)	0.770

The *p* values refer to the significance of a rank Wilcoxon statistics for dependent sample

*FA* femoral anteversion, *PCA* posterior condylar angle, *TEA-PTC* angle between transepicondylar surgical axis and tangent to the posterior prominent point of the medial and lateral tibial condyles, *TEA-PTT* angle between transepicondylar surgical axis and tangent to the posterior surface of the tibia at TT sections, TEA-TFA: angle between transepicondylar surgical axis and line joining the center of both malleoli, *AMA* anatomical–mechanical angle of femur, *FI* femoral inclination

^a^Single limbs were excluded in order to apply a rank test for dependent sample

**Table 2:** Median and percentiles for each variable considered stratified by side of the limb and gender of the patient

Panel A: right limb (*n* = 38)			
	Male (*n* = 20) Median (2.5%; 97.5%)	Female (*n* = 18) Median (2.5%; 97.5%)	*p* value
FA	10.9 (− 3.0; 25.0)	22.6 (5.0; 36.6)	0.003
PCA	2.8 (− 0.5; 4.5)	3.3 (1.0; 6.2)	0.044
TEA-PTC	0.1 (− 6.7; 9.2)	2.4 (− 2.2; 7.0)	0.260
TEA-PTT	− 1.2 (− 4.5; 2.8)	− 1.7 (− 10.9; 3.3)	0.578
TEA-TFA	29.6 (19.1; 41.2)	33.0 (15.0; 43.8)	0.465
PTC-TFA	28.6 (17.1; 44.1)	30.2 (16.3; 43.0)	0.619
AMA	4.8 (2.8; 6.6)	6 (3.9; 6.6)	0.009
FI	124.8 (119.0; 132.2)	125.8 (120.9; 132.9)	0.265

Panel B: left limb (*n* = 37)			
	Male (*n* = 19) Median (2.5%; 97.5%)	Female (*n* = 18) Median (2.5%; 97.5%)	*p* value
FA	19 (1.3; 28.1)	20.5 (9.3; 33.0)	0.194
PCA	3 (− 1.5; 5.7)	3 (0.0; 5.3)	0.532
TEA-PTC	1 (− 6.4; 7.6)	1.46 (− 5.4; 5.7)	0.483
TEA-PTT	− 1 (− 4.9; 3.1)	− 2.1 (− 10.3; 4.6)	0.438
TEA-TFA	30.1 (10.0; 39.7)	31.0 (12.4; 46.8)	0.395
PTC-TFA	26.2 (12.0; 44.0)	29.2 (12.9; 43.5)	0.236
AMA	4.6 (3.1; 6.0)	5.8 (2.9; 6.6)	0.012
FI	125.1 (120.8; 132.6)	125.7 (122.9; 134.6)	0.446

The *p* values refer to the significance of a rank Wilcoxon statistics for independent samples

F*A* femoral anteversion, *PCA* posterior condylar angle, *TEA-PTC* angle between transepicondylar surgical axis and tangent to the posterior prominent point of the medial and lateral tibial condyles, *TEA-PTT* angle between transepicondylar surgical axis and tangent to the posterior surface of the tibia at TT sections, *TEA-TFA* angle between transepicondylar surgical axis and line joining the center of both malleoli, *AMA* anatomical–mechanical angle of femur, *FI* femoral inclination

**Table 3:** Median and percentiles for each variable considered stratified by side of the limb and age of the patient

Panel A: right limb (*n* = 38)			
	Age ≤ 75 years (*n* = 18) Median (2.5%; 97.5%)	Age > 75 years (*n* = 20) Median (2.5%; 97.5%)	*p* value
FA	12 (4.9; 33.2)	13.4 (− 4.1; 33.4)	0.988
PCA	3 (0.4; 5.6)	3 (− 0.4; 5.7)	0.308
TEA-PTC	0.6 (− 6.8; 7.0)	1.9 (− 4.1; 9.2)	0.629
TEA-PTT	− 1.2 (− 3.6; 3.8)	− 1.9 (− 10.5; 2.2)	0.278
TEA-TFA	32.9 (16.3; 43.8)	28.1 (17.1; 40.0)	0.373
PTC-TFA	29.6 (16.7; 48.4)	28.5 (16.6; 41.5)	0.619
AMA	5 (3.7; 6.9)	5 (2.8; 6.3)	0.596
FI	125 (120.9; 132.4)	125.8 (119.0; 132.6)	0.318

Panel B: left limb (*n* = 37)			
	Age ≤ 75 years (*n* = 17) Median (2.5%; 97.5%)	Age > 75 years (*n* = 20) Median (2.5%; 97.5%)	*p* value
FA	20 (9.8; 28.0)	17.3 (1.3; 32.9)	0.751
PCA	3 (0.5; 5.7)	3 (− 1.4; 5.2)	0.397
TEA-PTC	2 (− 6.6; 5.8)	1 (− 4.4; 7.6)	0.976
TEA-PTT	− 2.5 (− 6.2; 3.1)	− 0.5 (− 9.3; 4.6)	0.103
TEA-TFA	31.1 (9.8; 40.4)	30.2 (14.6; 46.4)	0.796
PTC-TFA	28.6 (12.0; 44.6)	27.2 (11.3; 43.4)	0.784
AMA	5 (4.0; 6.2)	5 (2.5; 6.5)	0.655
FI	125 (120.7; 134.5)	125.8 (123.0; 132.5)	0.436

The *p* values refer to the significance of a rank Wilcoxon statistics for independent samples

*FA* femoral anteversion, *PCA* posterior condylar angle, *TEA-PTC* angle between transepicondylar surgical axis and tangent to the posterior prominent point of the medial and lateral tibial condyles, *TEA-PTT*: angle between transepicondylar surgical axis and tangent to the posterior surface of the tibia at TT sections, *TEA-TFA* angle between transepicondylar surgical axis and line joining the center of both malleoli, *AMA* anatomical–mechanical angle of femur, *FI* femoral inclination

**Fig. 4:**
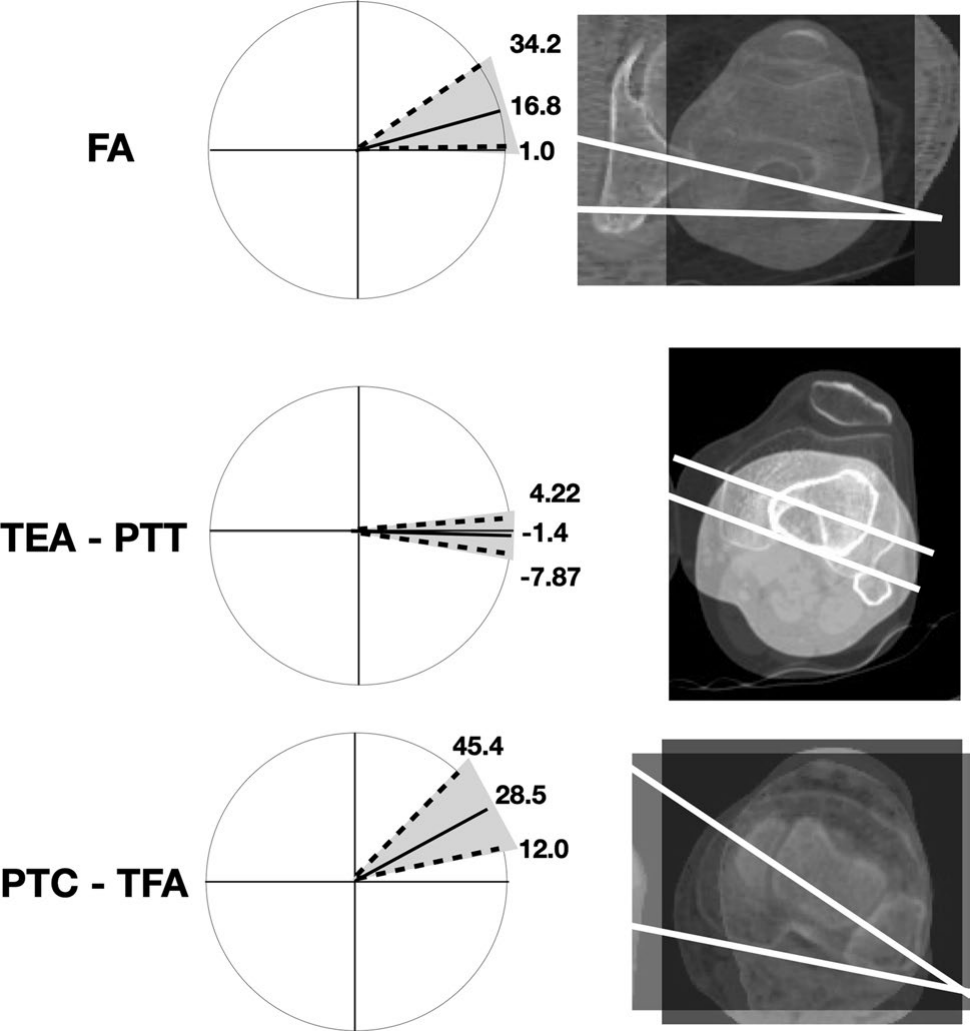
Graphic and CT representation of “normal” lower limb alignment observed in a Caucasian non-arthritic aged 36 subjects (left side). A wide variability was observed for each tomogram. Femoral torsion (FA) is measured as the angle between a line in the femoral neck axis and a line joining the posterior borders of both condyles. Femoral–tibial torsion (TEAs-PTT) is measured as the angle between transepicondylar surgical axes (TEAs) and a tangent to the posterior surface of the tibia at TT sections (PTT). Tibial torsion (PTC-TFA) is measured as the angle between a line joining the posterior borders of the tibial plateaus at the level of the tibial insertion of the posterior cruciate ligament and a line joining the center of the medial and the lateral malleolus. Dotted line: 2.5th and 97.5th percentile for each variable analyzed. Solid line: median of distribution of variable

Regarding axial alignment, the AMA was 5 (2.9; 6.8). No significance differences were reported by side. AMA was significantly lower in men in both the limbs. The FI was 125.3 (120.0; 134.8) with no statistically significant differences in terms of side age or gender. Regarding torsion alignment, the median values of FA, PTC-TFA and TEAs-PTT were, respectively, 16.8, 28.5 and − 1.4. Differences in terms of side, age and gender are summarized in Tables 2 and 3. A wide variability was observed for all tomogram’s measures in each patients’ group (Fig. 4).

## Discussion

The main contribution of the present study was to describe the torsion alignment of lower limb in an aged non-arthritic cohort. The study was carried out by CT which is considered the most accurate method to measure the lower limb alignment [13]. The anatomical landmarks used have been chosen by consensus of orthopedics and radiologists [19]. In contrast to other studies (Table 4), we selected only aged subjects without OA in order to consider a cohort that presumably will never develop idiopathic OA.

**Table 4:** Studies of axial and rotational alignment of lower limb in non-arthritic subjects

Authors	Year	Subjects (male %)	Age	Ethnic group	Method of measure	Statistics	FA	Tibial fibular torsion
Barahona et al. [13]	2019	32 (56%)	55 (median)	Caucasian (Chile)	CT	Interquantile (25 to 75)	9 (5 to 14)	30 (24 to 34)
Mullaji et al. [26]	2008	100 (84%)	26–40	Indian	CT	Mean ± SD	N/A	21.6 ± 7.6
Tamari et al. [23]	2006	25	> 60	Caucasian (Australia)	Inclinometer	Mean ± SD	12.7 ± 8.7	40.2 ± 8.4
		39		Japanese			16.1 ± 12.0	39.1 ± 11.1
Lang et al. [25]	1998	7	26–73	Caucasian	CT	Mean ± SD	N/A	33.14 ± 12.24 L 34.43 ± 8.16 R
Strecker et al. [24]	1997	355 (65%)	16–78	Caucasian (Germany)	CT	Mean ± SD	23.77 ± 18.27 (right side)	34.85 ± 15.85°
							24.46 ± 16.30 (left side)	
Reikerås et al. [28]	1989	50 (48%)	16–70	Caucasian (Norway)	CT	Mean ± SD	N/A	32.3 ± 8.5 F-R 30.7 ± 10.4 F-L 35.3 ± 7.6 M-R 34.0 ± 10.3 M-L
Clements et al. [27]	1988	100 (40%)	18–61	Caucasian (Sweden)	Fluoroscope	Mean ± SD	N/A	30.7 ± 5.2
Jend et al. [29]	1981	70	> 18	Caucasian (Germany)	CT	Mean ± SD	N/A	40 ± 9

*FA* femoral anteversion, *CT* computer tomography, *N/A* not applicable, *F* female, *M* male, *R* right, *L* left, *SD* standard deviation

It has been well established that coronal malalignment increases the risk of tibiofemoral OA [5]. Varus and valgus alignment shifts the load-bearing axis, respectively, medial and lateral to knee center, creating a moment arm that increases forces across the tibial plateau [1]. The median value of AMA measured in the present study was 5 (2.9; 6.8) that represents the “normal” and physiological alignment of lower limb with a load-bearing axis that runs through center hip, knee and ankle [20]. A possible limit of the present study was that we evaluated an axial non-weight-bearing alignment through CT. However, Paternostre et al. reported a not significance difference between weight and non-weight-bearing alignment in terms of varus and valgus [21].

The median value of FA measured in the present study was 16.8 (1.0; 34.2). By comparing the distributions of the angles among the sides, we found that only FA was significantly lower in the right limb. This difference has been reported also by previous studies [22], and by analogy, we did not find any reasonable explanation for this result. We also found that females presented a significantly higher FA in the right side (p. 0.003). It is interesting to note that “normal” FA here observed was characterized by a wide range of values in agreement with previous studies on healthy patients (Table 4) [13, 23, 24]. Therefore, a FA that differs from the median value might not be an indicator of pathological condition by itself. Further studies are necessary to considering the influence of FA in hip, knee or ankle pathologies.

The median value of tibiofibular torsion (PTC-TFA) measured in the present study was 28.5 degrees, which is in accordance with the values obtained from Caucasian limbs [24, 25] and markedly greater than the values obtained from Indian and Japanese subjects (Table 4) [26]. This difference could be related to genetic or cultural (i.e., gait and postural) differences. Right tibiae showed an increased median of external torsion of 1.5° compared with left tibiae (Table 1, *p* = 0.035); this agrees with most published data [24, 26–29], even though clinical significance of this right-left difference is not known.

In addition to current knowledge about axial and rotational alignment of human lower limb, in the present study, we analyzed the relationships between TEAs, PTC and PTT in healthy subjects. The median value of angle formed by TEAs and PTT was − 1.4 (− 7.9; 4.2) degree with no differences in terms of side and gender. These results suggest that the posterior tibial surface in correspondence of the proximal extremity of tibial tuberosity was normally slightly internally rotated in this cohort. This angle is original in the literature, and thus, it cannot be compared with measurements of other studies. This study had several limitations. First, inter-observer and intra-observer bias was not determined. However, previous studies reported excellent inter-rater reliability, suggesting that bias was minimal [13]. Although the sample size was underpowered to estimate normal values for the general cohort, there are no studies in the literature that analyze the rotational alignment in aged healthy cohort.

## Conclusion

In conclusion, the present study provides a detailed overview about the variability of axial and rotational alignment in Caucasian aged non-osteoarthritic cohort. The broad variability of the parameters analyzed highlights the necessity for a more anatomical and individualized approach during surgery of lower limb. The present study also offers the fundament to understand and treat lower limb deformities. Hence, these data can constitute the normal reference values useful to investigate lower limb malalignment. Moreover, it helps to assess in further studies the possible changes of axial and rotational alignment in idiopathic OA of lower limb.
